# Models for Prediction of Factor VIII Half-Life in Severe Haemophiliacs: Distinct Approaches for Blood Group O and Non-O Patients

**DOI:** 10.1371/journal.pone.0006745

**Published:** 2009-08-25

**Authors:** Kathelijn Fischer, Ronan Pendu, Carina J. van Schooten, Karin van Dijk, Cécile V. Denis, H. Marijke van den Berg, Peter J. Lenting

**Affiliations:** 1 Van Creveldkliniek, Department of Haematology, University Medical Center Utrecht, Utrecht, the Netherlands; 2 Julius Center for Health Sciences and Primary Care, University Medical Center Utrecht, Utrecht, the Netherlands; 3 Institut National de la Santé et de la Recherche Médicale (INSERM) U770 & Univ Paris-Sud, Le Kremlin-Bicêtre, France; 4 Department of Clinical Chemistry and Haematology, University Medical Centre Utrecht, Utrecht, the Netherlands; City of Hope Medical Center, United States of America

## Abstract

**Background:**

Von Willebrand factor (VWF) is critical for the *in vivo* survival of factor VIII (FVIII). Since FVIII half-life correlates with VWF-antigen pre-infusion levels, we hypothesized that VWF levels are useful to predict FVIII half-life.

**Methodology:**

Standardized half-life studies and analysis of pre-infusion VWF and VWF-propeptide levels were performed in a cohort of 38 patients with severe haemophilia A (FVIII <1 IU/ml), aged 15–44 years. Nineteen patients had blood-group O. Using multivariate linear regression-analysis (MVLR-analysis), the association of VWF-antigen, VWF-propeptide, age and body-weight with FVIII half-life was evaluated.

**Principal Findings:**

FVIII half-life was shorter in blood-group O-patients compared to non-O-patients (11.5±2.6 h *versus* 14.3±3.0 h; *p* = 0.004). VWF-antigen levels correlated with FVIII half-life considerably better in patients with blood-group non-O than O (Pearson-rank = 0.70 and 0.47, respectively). Separate prediction models evolved from MVLR-analysis for blood-group O and non-O patients, based on VWF-antigen and VWF/propeptide ratio. Predicted half-lives deviated less than 3 h of observed half-life in 34/38 patients (89%) or less than 20% in 31/38 patients (82%).

**Conclusion:**

Our approach may identify patients with shorter FVIII half-lives, and adapt treatment protocols when half-life studies are unavailable. In addition, our data indicate that survival of FVIII is determined by survival of endogenous VWF rather than VWF levels per se.

## Introduction

The bleeding disorder haemophilia A is caused by defects in the gene encoding coagulation factor VIII and affects 1–2 in 10,000 male births [1]. In plasma, FVIII circulates in a tight non-covalent complex with von Willebrand factor (VWF). The formation of this complex is of physiological importance to maintain appropriate plasma levels of FVIII [2], [3]. Indeed, patients lacking VWF (von Willebrand disease (VWD) type 3)) not only have a secondary deficiency of FVIII, but also a strongly reduced half-life of intravenously administered FVIII [4].

Current treatment of patients with severe haemophilia A (FVIII <1 IU/ml) mainly involves replacement therapy using purified plasma-derived or recombinant FVIII. Since the early 1970s, treatment protocols started to shift from on-demand replacement-therapy in cases of bleeds, towards prophylactic treatment to prevent bleeds. Initially, this was applied for patients who had already experienced several joint bleeds [5]. More recently, prophylactic treatment is administered more stringently and is often started before the onset of joint damage and after no more than 1 or 2 joint bleeds. Although prophylactic treatment has been proven to be beneficial for the patient, it requires regular infusions (up to 3 times per week) because of the relative short half-life of FVIII. The average half-life of FVIII in adult haemophilia A patients is 12 h, but a large variation between individuals (6–29 h) has been observed [6], [7]. Individualization of prophylactic treatment according to half-life may help optimizing treatment-protocols and improve efficiency of FVIII use. This is of particular relevance in children on prophylaxis, since bleeding is determined by time spent below 1% FVIII activity levels [8]. Also, information on FVIII half-life might be needed during a perioperative period of replacement-therapy in both children and adults, to determine the optimal interval between FVIII infusions and/or dosing during continuous infusion. However, individual half-life studies requiring frequent venipunctures during 72 h are time-consuming and difficult to perform especially in children [8].

Few factors have been identified that are associated with this variation in half-life. Of these, VWF levels appear most relevant: a correlation between pre-infusion VWF levels and FVIII half-life has been described in at least two separate studies [6], [9]. Since VWF levels are on average 25% lower in blood group O individuals compared to blood group non-O individuals, it is therefore not surprising that FVIII half-life is shorter in haemophiliacs with blood-group O than in those with other blood-groups [9], [10].

Recently, we have described a cohort of 38 severe haemophilia A patients, in whom FVIII half-life has been determined [7]. We have re-analyzed these data in order to investigate which parameters can be used to predict FVIII half-life in these patients with reasonable accuracy. In addition to VWF-antigen, we also considered VWF-propeptide (which is secreted simultaneously with mature VWF in a 1∶1 ratio) [11], the VWF/propeptide ratio (which provides information on the relative differences in clearance rates between both proteins) [12], [13], age and body-weight (both of which are known to influence VWF levels) [14].

## Materials and Methods

### Patients

From the cohort treated at the Van Creveldkliniek, thirty-eight patients with severe haemophilia A (FVIII activity <0.01 IU/ml) were included in the present analysis. All came from a cohort of 42 patients selected according to phenotype that has been described elsewhere [7], [15]. None of the patients were HIV-positive, had liver failure, fever in the previous 48 h, elevated CRP, low platelets, increased prothrombin time, low factor V, signs of liver cirrhosis on ultrasound, severe bleeding or surgery in the previous 3 months, or antibodies against FVIII (as assessed by an immunosorbent-assay and a Bethesta-assay (sensitivity 0.3 Bethesta Units/ml)). Mean age was 26.3 years (standard deviation 11.6, range 10.3–47.2). Four patients were excluded from the present study, because no plasma material was available for determination of VWF propeptide levels at the time of analysis, leaving 38 patients for this study. Nineteen patients were blood-group O, and nineteen were non-O (12 A, 5 B, 2 AB).

### Study design

The present analysis was based on data from a previous study, in which FVIII half-life was assessed in a standardized manner [7]. In short: patients had had no FVIII for at least 72 h before testing, they received a dose of 50 IU/kg recombinant FVIII concentrate, blood samples were taken at 15 min, 30 min and 1, 3, 5, 24, 30, 48 and 60 h after infusion. The pharmacokinetic program PK solutions (Summit Research Services, Montrose, CO) was used to calculate FVIII half-life using model-independent calculations, as recommended elsewhere [16].

### Laboratory assays

VWF-antigen and VWF-propeptide antigen levels were determined and values were converted into molar concentrations as described [11], [17], [18]. Antigen levels were determined independently, without knowledge of corresponding FVIII half-life values in order to improve internal validity.

### FVIII survival studies in FVIII-deficient mice

FVIII-deficient mice (mixed C57Bl6/J-129 Sv background with a deletion in exon 16, obtained from The Jackson Laboratory, Bar Harbor, ME) were between 8 and 10 weeks of age. Mice were treated with recombinant interleukin-11 (rIL-11, obtained from ProSpec, Rehovot, Israel) to raise VWF levels or vehicle as described previously [19]. Briefly, blood was first collected 3 days before injections started to determine base-line values. To this end, mice were anaesthetized using isoflurane, and blood was obtained via retro-orbital puncture. Two drops of blood were collected in EDTA (5 mM) for blood counts and 200 microliters were collected in citrate for VWF antigen determination. Subsequently, mice were injected subcutaneously with vehicle or rIL-11 (250 µg/kg) for 7 consecutive days. On day 8, mice were injected intravenously via the tail-vein with recombinant VWF-free FVIII (Kogenate, 150 U/kg). At indicated timepoints (3 min, 15 min, 30 min or 1 h), mice were anaesthetized using tribromoethanol (0.15 ml per 10 g of body weight). Blood was collected in a similar fashion (2 drops in EDTA for blood count and 200 microliters in citrate for VWF antigen and FVIII activity). VWF antigen and FVIII activity were determined as described previously [20], [21]. For each time-point three mice were used in each group.

### Ethics statement

All patients and/or parents gave written informed consent for the sampling of blood for scientific purposes, per the Declaration of Helsinki. Approval was obtained from the institutional review board of the University Medical Center Utrecht (Utrecht, the Netherlands). Animal housing and experiments were done as recommended by French regulations and according to experimental guidelines of the European Community.

### Data analysis

First, Pearson correlations for FVIII half-life with VWF-antigen, VWF-propeptide, VWF/propeptide ratios, age and body-weight were determined. Subsequently, stepwise-backwards multivariate linear regression (MVLR)-analysis was used to assess the independent associations of these parameters with FVIII half-life (SPSS 12.0 Inc, Chicago, IL). All parameters with a p-value of 0.15 or higher were excluded after each step. Separate analyses were performed for blood-group O and non-O. The stepwise-backwards procedure was used because all of the tested variables represent parameters known to be associated with VWF metabolism. To assess calibration of the models, FVIII half-lives predicted by the model were compared to the observed half-life for each patient. Absolute and proportional deviations from observed half-lives were calculated. Data are presented as mean±SD unless stated otherwise. P-values of less than 0.05 were considered statistically significant.

## Results

### FVIII half-life in blood-group O and non-O haemophilia A patients

The FVIII half-life in this cohort of 38 severe haemophilia A patients varied between 7.4 and 20.4 h, with a mean half-life of 12.9±3.1 h (mean±S.D.) [7]. Patient characteristics and test results according to blood-group are summarized in Table 1. As expected, average FVIII half-life was significantly shorter in blood-group O patients than in non-O patients (11.5±2.6 h *versus* 14.3±3.0 h). Furthermore, average VWF levels were lower in blood-group O patients than in non-O patients (36.2±7.0 nM versus 51.1±22.2 nM). In contrast, propeptide levels were similar for both groups (4.7±1.0 nM *versus* 5.0±1.6 nM). As a consequence, VWF/propeptide ratios were significantly increased in blood-group non-O patients compared to O patients (10.3±2.5 *versus* 7.8±1.5).

**Table 1:** Patient characteristics according to blood-group.

	Blood-group non-O	Blood-group O	*p*-value
	N = 19	N = 19	
Age (y)	28.1±10.0	27.5±13.6	0.88
Body-weight (kg)	75.8±18.4	67.1±18.4	0.15
FVIII half-life (h)	14.3±3.0	11.5±2.6	<0.01
VWF-antigen (nM)	51.1±22.2	36.2±7.0	<0.01
VWF-propeptide antigen (nM)	5.0±1.6	4.7±1.0	0.61
VWF/propeptide ratio	10.3±2.5	7.8±1.5	<0.01

Data represent mean±SD.

### Pre-infusion VWF-antigen levels and FVIII half-life

**Figure 1:**
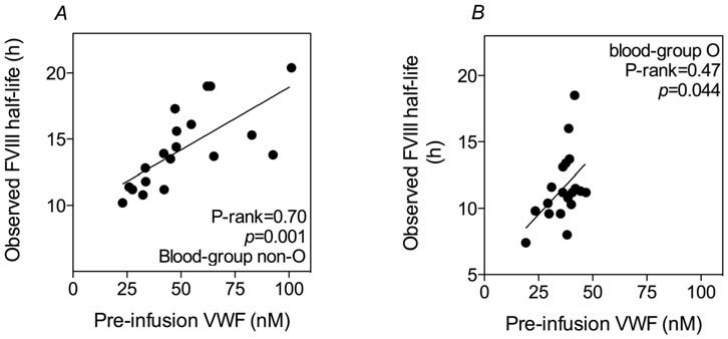
Relation between FVIII half-life and pre-infusion VWF-antigen levels. Panels show the correlation between FVIII half-life and VWF pre-infusion levels in severe haemophiliacs with blood-group non-O (*panel A*) and blood-group O (*panel B*). Drawn lines represent best-fit obtained after univariate linear regression.

In view of the previously reported correlation between VWF pre-infusion levels and FVIII half-life [6], it was of relevance to determine if this correlation is dependent on blood-group. A significant correlation between pre-infusion VWF-antigen levels and FVIII half-life was observed in blood-group non-O patients (Pearson-rank = 0.70; *p* = 0.001; Fig. 1A). However, this correlation appeared to be less obvious in O-patients, although it still reached statistical significance (Pearson-rank = 0.47; *p* = 0.044; Fig. 1B). Given these blood-group dependent differences, the association between the various parameters and FVIII-half-life was evaluated separately for blood-group O and other patients.

### Univariate regression-analysis of parameters potentially useful for prediction of FVIII half-life

In search for additional valuable parameters to predict FVIII half-life, we considered a number of alternatives besides VWF-antigen: *(1)* VWF-propeptide, which is secreted in a 1∶1 molar ratio with VWF. In contrast to mature VWF, VWF-propeptide is devoid of ABO-glycan determinants [12]; *(2)* VWF/propeptide ratio. Due to differences in half-life of mature VWF and VWF-propeptide, a distinct VWF ratio is present under steady-state conditions [13]. A more or less pronounced clearance of VWF compared to its propeptide will become apparent via this ratio; *(3)* Age and body-weight, both of which may influence VWF-antigen levels [14].

In a first approach, Pearson correlations for each separate parameter were determined, the results of which are summarized in Tables 2 and 3. For blood-group non-O, a strong correlation with FVIII half-life was observed for VWF propeptide (Pearson-rank = 0.76; *p* = 0.001), which appeared to be slightly better compared to VWF-antigen. A weaker correlation was observed for age (Pearson-rank = 0.50; *p* = 0.028). For blood-group O patients, a strong correlation with FVIII half-life was observed for VWF/propeptide ratio only (Pearson-rank = 0.70; *p* = 0.001), which was considerably stronger than the correlation found for VWF-antigen.

**Table 2:** Results of univariate linear regression analysis blood-group non-O patients.

Determinant	Regression coefficient	95% CI	*p*-value
Age (y)	0.15	0.02 to 0.29	0.03
Body-weight (kg)	0.02	−0.06 to 0.11	0.56
VWF-antigen (nM)	0.10	0.05 to 0.14	<0.01
VWF-propeptide antigen (nM)	1.41	0.80 to 2.02	<0.01
VWF/propeptide ratio	0.28	−0.33 to 0.89	0.34

**Table 3:** Results of univariate linear regression analysis blood-group O patients.

Determinant	Regression coefficient	95% CI	*p*-value
Age (y)	0.04	−0.06 to 0.14	0.42
Body-weight (kg)	0.03	−0.04 to 0.10	0.41
VWF-antigen (nM)	0.18	0.01 to 0.34	0.04
VWF-propeptide antigen (nM)	−0.54	−1.92 to 0.83	0.42
VWF/propeptide ratio	1.19	0.57 to 1.82	<0.01

### Stepwise-backwards MVLR-analysis

**Figure 2:**
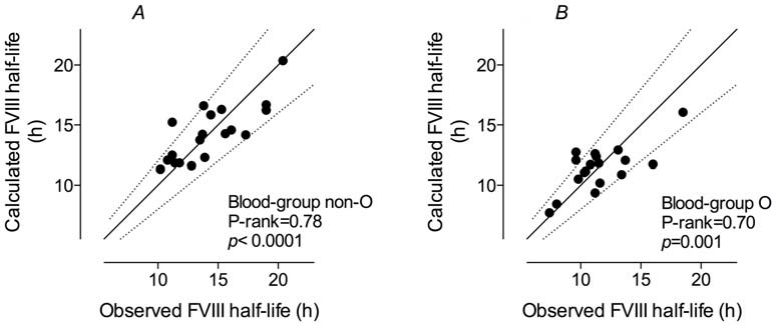
Calculated FVIII half-life versus observed FVIII half-life. Calculated FVIII half-lives were obtained using equations 1 or 2 (*Panels A* and *B*, respectively), using pre-infusion VWF and propeptide levels, and are plotted versus observed FVIII half-lives of blood-group non-O (*panel A*) and blood-group O patients (*panel B*). Solid lines represent ideal correlation between calculated and observed half-lives, while dotted lines indicate 20% deviation margins.

In a subsequent step, data were analyzed via stepwise-backwards MVLR-analysis to assess interdependence of these parameters. In each step, each parameter having a *p*-value of more than 0.15 was eliminated. Using this approach, age, weight and propeptide were eliminated as predictive parameters while analyzing blood-group non-O patients. The remaining parameters (VWF-antigen and VWF/propeptide ratio) were used to define the following predictive equation, which explained 57% of all variability in FVIII half-life (see Fig. 2A):
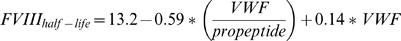
(1)


For blood-group O patients, the sole predictive parameter that remained following stepwise-backwards MVLR-analysis was the VWF/propeptide ratio, resulting in predictive equation 2. Equation 2 is able to explain 49% of all variability in FVIII half-life in this patient group (see Fig. 2B).
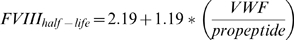
(2)Model performance of MVLR-analysis according to blood-group is shown in Tables 4 and 5. The median deviation was +0.30 h (95% CI from −0.87 to +0.93) for blood-group non-O patients and +0.40 h (95% CI from −0.92 to +0.87) in patients with blood-group O. Thus, there is a slight tendency to overestimate FVIII half-life using these predictive equations. Nevertheless, the overall predicted half-life was within 3 h difference of the observed half-life in 34 of 38 patients (89%), when comparing predicted and observed half-lives. The proportional difference was within 20% in 31 of 38 patients (82%).

**Table 4:** Results of multivariate linear regression analysis blood-group non-O patients.

Determinant	Regression coefficient	95% CI	*p*-value
VWF-antigen (nM)	0.14	0.08 to 0.20	<0.01
VWF/propeptide ratio	−0.59	−1.15 to −0.04	0.04

**Table 5:** Results of multivariate linear regression analysis blood-group O patients.

Determinant	Regression coefficient	95% CI	*p*-value
VWF/propeptide ratio	1.19	0.57 to 1.82	<0.01

When analyzing the distribution of half-lives in our cohort, it appeared that the majority of the patients (27 of 38, *i.e.* 71.0%) displayed half-lives between 10 and 14 h. Only 2 patients (5.3%) had a half-life of less than 10 h, whereas 9 patients (23.7%) had a FVIII half-life longer than 14 h. To test whether our model would be useful to identify patients that have half-lives outside the range of 10–14 h, we examined how many patients where correctly placed in either of the three groups (<10, 10–14, >14 h). We found that 31 of 38 patients (82%) were correctly grouped in each of the three groups after applying our model. Of the 7 wrongly grouped patients, 3 patients were predicted to have a half-life >14 h, whereas their observed half-life was 10–14 h. Three other patients had a FVIII half-life >14 h, but where predicted to be in the 10–14 h group. One patient with an observed half-life of 10 h was predicted to have a half-life of 9 h.

### Effect of raising VWF levels in vivo on FVIII survival

Given the strong relationship between VWF pre-infusion levels and FVIII half-life, it was of interest to investigate whether manipulation of VWF levels is a useful tool to influence FVIII survival. Two potential mechanisms may explain this strong relationship. First, individual VWF levels are determined by its clearance rate, in which low clearance is associated with increased VWF levels. In this scenario, FVIII would survive longer in the circulation in these individuals, since its carrier-protein VWF has a low clearance rate. Second, a larger excess of VWF over FVIII may force unbound FVIII molecules (normally estimated to encompass 2–5% of the FVIII molecules [22], [23]) into complex with VWF, thereby reducing the amount of free FVIII which is cleared more rapidly than VWF-bound FVIII. To investigate whether increasing VWF levels within the physiological range is sufficient to increase the survival of FVIII, we made use of the previous observation that treatment with rIL-11 is associated with an increase in VWF levels without affecting its half-life [19], [24], [25]. Therefore, FVIII-deficient mice were injected subcutaneously with rIL-11 (0.25 mg/kg) once a day for 7 consecutive days [19]. As expected, an increase in platelet count was observed for rIL-11-treated mice (478±59 10^3^ platelets/µl and 708±81 10^3^ platelets/µl, before and after treatment; mean±S.D.) but not for placebo-treated mice (472±90 10^3^ platelets/µl and 483±144 10^3^ platelets/µl, before and after treatment). Also VWF levels were increased upon rIL-11 treatment, resulting in levels that were 1.7-fold higher in rIL-11-treated *FVIII-*null mice compared to placebo-treated *FVIII*-null mice. At day 8, both rIL-11-treated and placebo-treated mice were injected intravenously with recombinant FVIII (150 U/kg) and blood was collected up to 1 h after injection (3 mice per time-point for each group) to determine FVIII survival. Analysis of FVIII activity in plasma samples revealed no difference in residual FVIII activity at any of the time-points tested (up to 1 hour after injection; see supplementary Figure S1). Thus, increasing steady-state VWF levels was not associated with an increased survival of FVIII in this mouse model.

## Discussion

It has been recognized since long that survival of intravenously administered FVIII varies greatly between haemophilia A patients. Individual half-life studies could therefore be used to optimize individual treatment protocols. Such studies often require multiple blood samplings over a period of several days. In order to simplify the issue of FVIII half-life determination, the current study presents blood-group specific prediction models based on the analysis of a cohort of 38 haemophilia A patients. This cohort consists of 19 patients having blood-group O and 19 patients having blood-group non-O. Since FVIII half-life differed significantly between both patient-groups (11.5±2.6 h versus 14.3±3.0 h, respectively), we decided to analyze both groups separately.

It has previously been reported that VWF pre-infusion levels correlate to some extent with FVIII half-life [6], [9], which was confirmed in our study. In order to build a prediction model, we also considered a number of parameters that are related to the metabolism of VWF. Via stepwise-backwards MVLR-analysis, only two parameters appeared to be of relevance with regard to their predictive value of FVIII half-life: VWF-antigen (for blood-group non-O patients) and VWF/propeptide ratio (for patients of both blood-groups). Using these parameters, FVIII half-life could be predicted with a deviation of maximal 3 h of the observed half-life in 34/38 patients (89%). This is a considerable improvement compared to the use of VWF antigen levels as a predictive parameter, which results in a deviation of maximal 3 h in 22/38 patients (58%). In addition, our analysis allowed us to identify those patients that have half-lives that are outside the normal range found in the majority of patients (less than 10 h or longer than 14 h) with an accuracy of >80%.

The finding that the inclusion of VWF-antigen levels in liaison with VWF/propeptide ratio was required for optimal prediction of FVIII half-life in non-O patients but not in blood-group O patients is intriguing. One potential explanation could originate from the larger variability in VWF-antigen levels in blood-group non-O patients (ranging from 23 to 101 nM) compared to blood-group O patients (ranging from 19 to 47 nM). Since VWF and its propeptide are secreted in a coordinated manner, the variability in VWF levels may be compensated via inclusion of propeptide levels. Indeed, the range in propeptide levels was merely overlapping in both patient groups (2.7–8.5 nM and 3.4–7.3 nM for patients with blood-group non-O and O, respectively).

Irrespective of the differences in the equations that describe the correlation with FVIII half-life between both patient groups (blood-group non-O versus O), we would like to emphasize that our observations are of an empirical character. Nevertheless, we feel that our approach has resulted in a simple method that allows prediction of FVIII half-life with reasonable accuracy and identification of patients with shorter half-lives. This approach may facilitate the design of dosing strategies for haemophilia A patients in the absence of individual half-life studies. It should be noted that our current study lacks a true verification of our model, since no second independent dataset has been tested. Additional studies are therefore needed to validate and, eventually to refine our findings.

Does the finding that increased VWF levels positively associate with FVIII half-life allows the conclusion that the survival of FVIII may be prolonged by artificially increasing endogenous VWF levels? This would be true if the effect of VWF is independent of the rate by which endogenous VWF is cleared from the circulation. Rather it should rely on the molar excess of VWF over FVIII, which theoretically would result in more FVIII being forced into complex formation with VWF. We have tested this possibility in an experimental mouse model, in which we used rIL-11 to increase steady-state levels of VWF over a prolonged period of time. However, FVIII administered to these mice displayed similar survival as FVIII injected in vehicle-treated control mice. The possibility exists that the lack of effect on FVIII half-life is because VWF levels were outside the range of correlation between VWF levels and FVIII half-life. However, VWF levels were increased in rIL-11 treated mice from 100% to 170%. This concentration is well within the range of correlation observed in our cohort, where VWF levels varied between 40% and 220% of normal. An alternative explanation could relate to the extent of VWF∼FVII complex formation, in that our system is too insensitive to detect the effect of a minor increase in complex formation on FVIII half-life. It can be calculated that an increase of 95% complex formation to 98% complex formation theoretically results in a 2.5–3% slower clearance of the FVIII molecule per 10 min in our mouse model (assuming mean residence time of 10 min for free FVIII and 150 min for VWF-bound FVIII). Over the 60-min period that we monitored, the accumulative difference in clearance should have been detectable in our experimental model. Since this was not observed, the extent in increase in VWF levels in our model is apparently too small to influence complex formation so that FVIII survival is affected.

The effect of increasing pre-infusion VWF levels on FVIII half-life has also been examined in human patients. One way to increase VWF levels is via treatment using desmopressin [26], [27]. Previously, two reports describing the effect of desmopressin-treatment on FVIII survival have been published. First, a study published in 1985 by McLellan and co-workers revealed no effect on FVIII survival upon treatment with desmopressin [28]. It should be noted that this study dates from over 20 years ago. It cannot be excluded that FVIII concentrates that were used in that study contained large amounts of VWF and therefore any increase in endogenous VWF may become less relevant. In a more recent study, Deitcher and colleagues compared plasma-derived and recombinant FVIII preparations that are essentially free of VWF (*i.e.* Monoclate-P and Bioclate) [29]. They reported similar half-lives for both preparations upon co-treatment with desmopressin. A similar result (no increase in half-life) was also reported in a preliminary study by Fijnvandraat and coworkers, in which a small group of patients was treated with Aafact (a plasma-derived FVIII therapeutic similar to Monoclate-P) in the presence or absence of desmopressin [30]. Apparently, increasing VWF-antigen via desmopressin- or rIL11-treatment is not helpful in prolonging half-life of FVIII.

In conclusion, FVIII half-life after infusion with recombinant FVIII concentrates in patients with severe haemophilia A is largely dependent on blood group. For blood-group non-O patients, FVIII half-life was best predicted by a model depending on both VWF antigen levels and on VWF/propeptide ratio. For patients with blood-group O, FVIII half-life was best predicted by VWF/propeptide ratio. The importance of the VWF/propeptide ratio in both models suggests that FVIII half-life is more dependent on the rate of endogenous VWF clearance rather than on absolute VWF levels. This possibility was supported by our mouse-model in which VWF levels but not FVIII survival was increased using rIL-11. The prediction-models presented may facilitate individualized treatment according to pharmacokinetics in the absence of individual half-life studies in haemophilia A patients.

## Supporting Information

Figure S1 Factor VIII-deficient mice were treated with vehicle or recombinant IL-11 for 7 consecutive days. VWF levels remained unchanged in vehicle-treated mice, but were increased 1.7-fold in IL-11 treated mice. Mice were subsequently injected intravenously with recombinant VWF-free FVIII (150 U/kg), and blood samples were taken at indicated time-points. The graph displays residual FVIII levels at indicated time-points for both vehicle- and IL-11 treated mice. At none of the indicated time-points, a statistical significant difference in residual FVIII activity was detected. Data represent mean±SD of 3 mice per time-point.
(0.03 MB PDF)
