# Supplementary material for: Models for Prediction of Factor VIII Half-Life in Severe Haemophiliacs: Distinct Approaches for Blood Group O and Non-O Patients
Source: PLoS One. 2009 Aug 25;4(8):e6745. doi: 10.1371/journal.pone.0006745 (PMC2727052; doi:10.1371/journal.pone.0006745)
Supplement: Figure S1 — Factor VIII-deficient mice were treated with vehicle or recombinant IL-11 for 7 consecutive days. VWF levels remained unchanged in vehicle-treated mice, but were increased 1.7-fold in IL-11 treated mice. Mice were subsequently injected intravenously with recombinant VWF-free FVIII (150 U/kg), and blood samples were taken at indicated time-points. The graph displays residual FVIII levels at indicated time-points for both vehicle- and IL-11 treated mice. At none of the indicated time-points, a statistical significant difference in residual FVIII activity was detected. Data represent mean±SD of 3 mice per time-point. (0.03 MB PDF) [file pone.0006745.s001.pdf]

Fischer et al.
Prediction models for factor VIII half-life in severe haemophiliacs: distinct approaches for blood group O and non-O patients

Supplementary figure

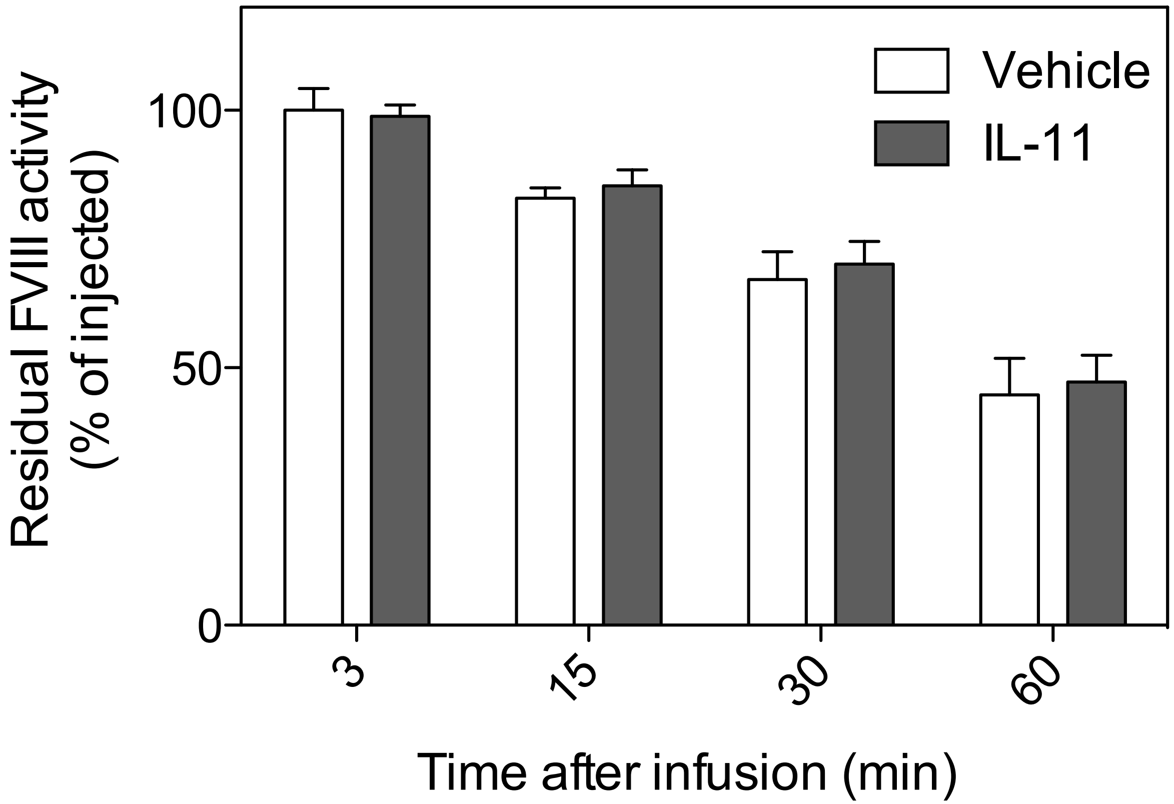

Legend: Factor VIII-deficient mice were treated with vehicle or recombinant IL-11 for 7 consecutive days. VWF levels remained unchanged in vehicle-treated mice, but were increased 1.7-fold in IL-11 treated mice. Mice were subsequently injected intravenously with recombinant VWF-free FVIII (150 U/kg), and blood samples were taken at indicated time-points. The graph displays residual FVIII levels at indicated time-points for both vehicle- and IL-11 treated mice. At none of the indicated time-points, a statistical significant difference in residual FVIII activity was detected. Data represent mean±SD of 3 mice per time-point.
